# The Effect of Acetic Acid as a Solvent on the Structure and Properties of Poly(3-hydroxybutyrate)—Based Dried Gels

**DOI:** 10.3390/gels10100664

**Published:** 2024-10-17

**Authors:** Vsevolod Zhuikov, Yulia Zhuikova

**Affiliations:** Research Center of Biotechnology of the Russian Academy of Sciences, 33, bld. 2 Leninsky Ave., 119071 Moscow, Russia; zhuikova.uv@gmail.com

**Keywords:** biodegradable polymers, PHB, gels, rheology, cytocompatibility, DSC, SEM, porosity

## Abstract

Poly(3-hydroxybutyrate) (PHB) is a microbially derived polyhydroxyalkanoate that is widely used in biomedical applications. In this study, we investigated the use of acetic acid (aa) as an alternative environmentally friendly solvent for the preparation of gels from PHB (PHB aa) and compared their characteristics with PHB products dissolved in chloroform (PHB chl) using such methods as DSC, FTIR, SEM, rheometry, biodegradation, and cytocompatibility assay. A slight decrease in the degree of the crystallinity of the PHB from 61% to 50.8% was found when the acetic acid was used. This resulted in a greater mass loss for the PHB aa (11%) during enzymatic degradation over 180 days. Gels prepared from PHB in the different solvents showed differences in the microstructure and porosity of the samples, which affected their viscoelastic properties. The storage modulus (G′) for the PHB aa gels was higher by 35% compared to that for the PHB chl, and Young’s modulus in compression was 101.5 and 41.3 kPa for the PHB aa and PHB chl, respectively. The porosity of the PHB aa was 97.7%, which was 5.2% higher than that for the PHB chl. The presence of low molecular weight polymers in the PHB aa had an effect on mesenchymal stem cells’ viability, expressed as a threefold increase in the number of attached cells after 7 days of incubation compared to the PHB chl. Thus, the proposed method of PHB-based materials’ preparation is a promising, more environmentally friendly analog of the extensively used method of preparation from chloroform.

## 1. Introduction

Modern life cannot be imagined without the widespread use of polymeric materials, which are used in all aspects of human life [1,2,3]. Synthetic polymers such as polyethylene, polypropylene, polyterephthalate, polyurethane, etc. [4,5,6,7], are much more widely used and studied than polymers of natural origin (polyhydroxyalkanoates, polysaccharides). One of the reasons for this is the variability of the structure and the difficulty of regulating the properties of biopolymers to obtain materials with uniform parameters, since the properties of biopolymers can vary depending on their origin, method of production, and purification [8,9]. Nevertheless, the study of biopolymers and structures based on them is an urgent task for a large number of researchers worldwide. This is primarily due to an important property of polymers of natural origin: the ability to degrade in a relatively short time into components that, unlike many synthetic polymers, are non-toxic to the human body and harmless to the environment due to their destruction in biological processes [10]. This makes it possible to use biopolymers in food [11,12], packaging [13,14], wastewater treatment [15], and agricultural industries [16], as well as in the manufacturing of implantable medical devices [17,18].

One of the most promising biopolymers is polyhydroxybutyrate (PHB) [19,20]. PHB is a polyester of microbial origin that belongs to the class of polyhydroxyalkanoates. A very important advantage over synthetic polymers is its ability to degrade into carbon dioxide and water under the action of enzymes [21,22,23]. This ability is a consequence of the fact that PHB is a storage substance for bacteria (*Azotobacter* sp., *Bacillus* sp., *Ralstonia* sp., etc.) and can also be found in animals [24]. In the medical field, it is equally important that PHB products have sufficient biocompatibility, including hemocompatibility [25], and low toxicity. However, several properties of PHB, such as its high degree of crystallinity [26], hydrophobicity, lack of water absorption, and long biodegradation time, can limit its widespread use [27]. Typically, a variety of copolymers, composites, and blends are prepared with both low molecular weight substances and other polymers, including those of natural origin, to modify and improve their properties, including physicochemical and biological properties [28,29]. PHB and its copolymers are used to make sutures, endoprostheses, matrices, coatings, gels for replacing defects in various tissues and cell cultures, drug delivery, controlled release systems, and much more [30,31,32,33].

There are various ways to create 3D materials with a high degree of porosity, such as leaching, freeze-drying, electrospinning, and others. The traditional method of forming 3D structures for PHB is the chemical leaching method [34]. One of the disadvantages of this method is the need for the additional introduction of pore formers. There are also studies on the formation of PHB matrices and gels by phase separation. These include the non-solvent-induced phase separation (NIPS) method [35], which consists in inducing phase separation by introducing a non-solvent into the polymer solution. PHB organogels have been obtained by this method, and aerogels have been made using freeze-drying processes [36,37]. Thermally induced phase separation (TIPS) is a physical method of forming gels, in which exposure to temperature causes the formation of microcrystals in a polymer-rich phase. In this process, the entire solution becomes a gel as a three-dimensional network is formed due to the microcrystals. According to an earlier study [38], PHB gels are formed by dissolving small amounts of PHB in a hot solution. While cooling below the gel melting temperature, the gelator self-assembles into 3D networks that immobilize the complete volume of the solvent, supporting their own weight without collapsing the gel structure. Thermally reversible PHA-based materials, in particular PHBV, were studied in [39], where transparent solutions obtained by dissolving PHBV in hot toluene became gels after cooling to room temperature. It was also observed that this process was completely reversible. The aerogels of the PHB were prepared by freeze-drying the thermoreversible gels, and the films were prepared by hot pressing the PHB pellets [33].

The most commonly used solvent for PHB is trichloromethane. Dichloroethane, 1,1,1,3,3,3-hexafluoroisopropanol, dimethylsulfoxide, and trifluoroacetic acid are also used [40,41]. However, while these solvents are well suited for dissolving PHB, they are themselves hazardous, often carcinogenic, and highly cytotoxic if left in the gels, and their production can lead to environmental degradation. Thus, researchers may be faced with the question of the feasibility of using a biomaterial that is safe for the environment and organisms if its widespread use is limited by the problem of the environmental friendliness of the solvents used [42]. One way to solve this problem is to find and use alternative and safer solvents [43]. Different solvents for poly(3-hydroxybutyrate) were evaluated by the calculation of the three-dimensional solubility parameters defined by Hansen [44]. It has been shown that acetic acid is categorized as a partial solvent. This means that it is capable of limited solubilization of PHB without heating. However, it is important to note that the crystalline component of PHB does not obey this solubility concept. The crystalline portion of PHB can render the polymer insoluble in most solvents at room temperature.

Acetic acid is widely used in the food, chemical, and pharmaceutical industries, and it is a component of some drugs. It is not a carcinogen or mutagen and can normally be formed in living organisms, including the human body, during biochemical processes. The authors of [45] were among the first to demonstrate that PHB can dissolve in acetic acid when heated for 30–60 min. However, the properties of the resulting samples are dependent on the heating temperature of the mixture. The potential hydrolysis of PHB polymer chains by acetic acid has not been thoroughly investigated.

The aim of our study was to modify the method of using acetic acid to dissolve PHB and obtain dried gels from it, as well as to compare the main physicochemical and biological properties of the materials prepared using chloroform and acetic acid as solvents.

## 2. Results and Discussion

An important consideration in the preparation of polymer products is the choice of solvent. Chloroform is the most commonly used solvent in the production of poly(3-hydroxybutyrate) products. It is a toxic, volatile, colorless liquid with a strong smell. Some research shows that when chloroform is used for the production of polymer products, part of the solvent remains in the polymer structure and can have a toxic effect on cell growth [45]. Glacial acetic acid can serve as an eco-friendly substitute for chloroform. It is an organic acid that is naturally synthesized in living organisms [46]. Therefore, if residual acetic acid molecules are present in polymeric products, they will not be harmful to living systems since they can be incorporated into the organism’s metabolic system. Acetic acid is not an optimal solvent for poly(3-hydroxybutyrate). Therefore, to dissolve the polymer, it is necessary to heat the acid to its boiling point of 118 °C. Anbukarasu et al. were among the first to use acetic acid as a solvent for PHB, and in their work, the dissolution time ranged from 40 to 60 min [47]. PHB pellets were used for the dissolution; however, we hypothesize that this treatment increases the degree of crystallinity of the homopolymer, making it difficult to dissolve. In our work, we also used acetic acid as a solvent. However, the polymer was pulverized to accelerate the dissolution. This resulted in a significantly reduced dissolution time of 1–5 min

### 2.1. Gel Preparation

In practical applications like biomedicine, 3D structures are preferred. For instance, in tissue engineering, the volumetric shape of the product may offer critical advantages over flat films due to the presence of an internal structure that affects the viability of cell cultures. The fabrication of 3D structures (gels) from PHB using chloroform traditionally requires the use of additional substances as pore-forming agents, such as ammonium bicarbonate [34]. However, using acetic acid as a solvent for PHB eliminates the need for a pore former and simplifies the gel fabrication process.

**Figure 1:**
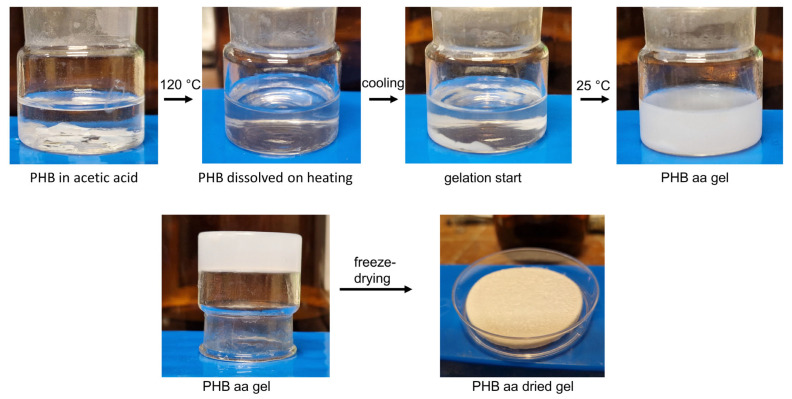
Gel preparation procedure in acetic acid.

The PHB aa was prepared as follows: PHB pellets were cut into small pieces and placed in a glass bouquet. Then, glacial acetic acid was added to the cut pellets. The solution was brought to a boil, which caused the polymer pieces to dissolve. When heated, the polymer solution in the acetic acid became transparent (Figure 1). The solution was then cooled at room temperature, during which it underwent a gelation process, forming an opaque gel. It is a system that consists of a three-dimensional polymer frame, the voids of which are filled with a low molecular weight organic solvent: acetic acid. For the dried gels, the solution was poured into a Petri dish, cooled, frozen at −20 °C, and lyophilized.

We assume that the gel formation occurred by the thermally induced phase separation method. In the case of semi-crystalline polymers, to which PHB belongs, crystallization occurs in the polymer-enriched phase as the temperature decreases. The solution gels as a three-dimensional network is formed due to microcrystals [36]. Thus, PHB gels in acetic acid are physical organogels. The authors of [38,48] suggested that the crystals formed during gelation are of the lamellae type. Several polymer chains are physically trapped within the crystal. As the solution cools, the chains form crystalline lamellae that serve as reversible cross-links if the entanglement of the polymer chains in the solution is sufficient.

### 2.2. Change in Molecular Weight of PHB aa

**Figure 2:**
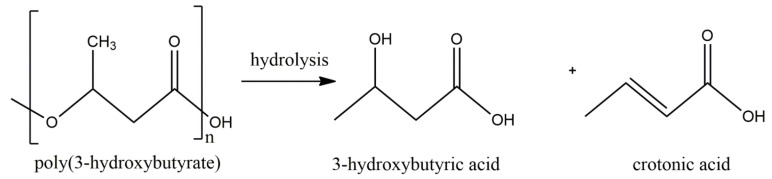
Formulae of polymer decomposition under the action of acid.

The main difference between our method and those previously described is the rapid dissolution time of the polymer. This is very important because, for PHB, acetic acid is the agent in the presence of which acid hydrolysis of the polymer will occur (Figure 2).

**Figure 3:**
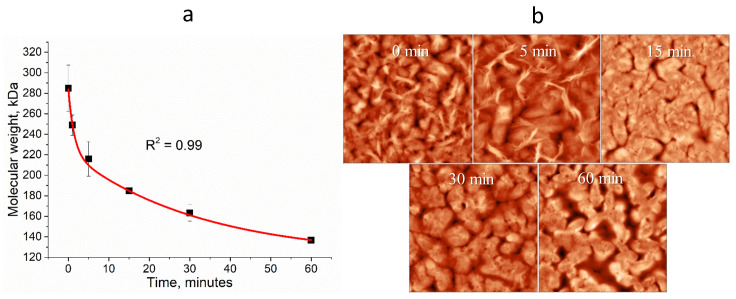
Kinetics of PHB molecular weight change in acetic acid solution for 1 h (**a**); AFM images of PHB films at different incubation times in acetic acid, scan area size 10 × 10 μm (**b**).

Based on this, an experiment was conducted to investigate the change in the molecular weight (MW) of the polymer dissolved in acid for 60 min. The polymer was dissolved in acetic acid at 120 °C. To measure the MW of the PHB in the acetic acid, 2 mL of the solution was taken at 0, 5, 15, 30, and 60 min after dissolution. The solution was then cooled and dissolved in chloroform to measure its viscosity using an Ubbelohde viscometer. The MW was calculated using the Mark–Hauvinck–Kuhn equation [49]. The resulting curve (Figure 3a) shows the decrease in the MW over time.

The data on the molecular weight decrease were fitted using a reaction rate equation consisting of two exponential terms. This is because the reaction rate exhibits a biphasic behavior. In the initial stage of hydrolysis, there is a sharp decrease in molecular weight. The decomposition rate constant for the first stage is calculated to be k1 = 0.71. The second stage of decomposition displays a gradual slope with a reaction rate constant of k2 = 0.03. This means the initial hydrolysis stage occurs nearly 24 times faster than the subsequent stage. This two-step hydrolysis phenomenon has also been observed in other polymers [50].

**Figure 4:**
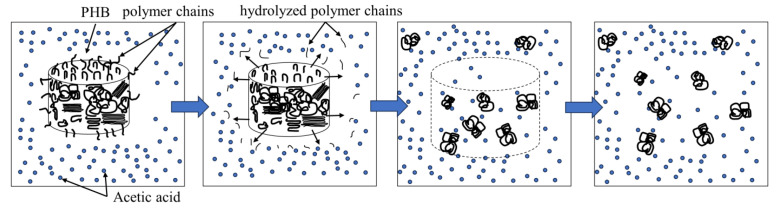
A probable explanation for the two-stage decrease in MW.

A possible explanation for this behavior is the following mechanism: when placed in acetic acid, the polymer is a compact solid with free polymer chains on its surface, as shown on Figure 4. These chains can be hydrolyzed upon exposure to acetic acid, leading to a decrease in MW. The subsequent swelling of the polymer causes the polymer network to diverge, which further increases the number of free polymer ends that can be rapidly hydrolyzed. This may account for the rapid decrease in the MW at the onset of dissolution. The decrease in the degradation rate may be due to the increase in the number of polymer particles during degradation, which leads to an increase in the polymer/acid ratio and a subsequent decrease in the hydrolysis rate. Additionally, acetic acid is a poor solvent for PHB, causing the polymer tangle to remain in a compact conformation, reducing the area available for the hydrolysis of the polymer chain [44,51,52,53].

In addition, PHB films were visualized by AFM during the acid hydrolysis process. The results are shown in Figure 3b. A gradual change in the morphology of the PHB samples can be seen. For the film studied immediately after dissolution (0 min), spherulites with surfaces covered by elongated fibers were observed. After 5 min, the fibrous areas became smaller and after 15 min they were not observed on the surface of the films. After 30 and 60 min, there was a gradual degradation of the spherulites expressed as a decrease in their size, probably due to the hydrolysis of the free polymer ends mentioned above. The experiment demonstrates that acetic acid can effectively serve as a solvent for PHBs during short heating times and can also control the reduction in the PHB’s molecular weight during longer incubation times. In our subsequent experiments, we aimed to prevent a significant reduction in the polymer’s molecular weight. Therefore, the samples under investigation were prepared by incubating the polymer in acetic acid for the shortest possible time (less than 1 min). The loss of the molecular weight during dissolution was assumed to be 15%.

### 2.3. Differential Scanning Calorimetry (DSC)

**Figure 5:**
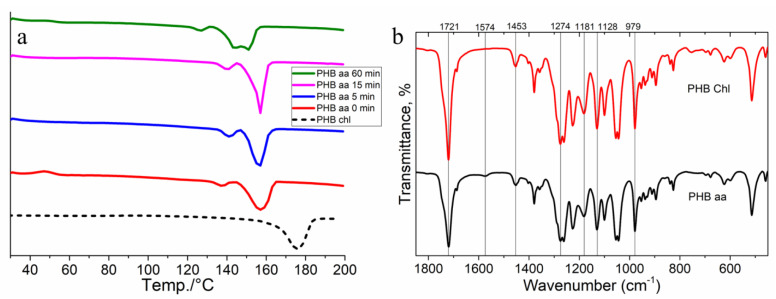
DSC thermograms of PHB from acetic acid with dissolution time 0, 5, 15, and 60 min and chloroform (**a**); FTIR spectroscopy for PHB chl and PHB aa (**b**).

The thermal properties of poly(3-hydroxybutyrate) as a function of the decomposition time in acetic acid were investigated. Figure 5 shows DSC thermograms of the samples obtained by dissolving the polymer in acetic acid for 0, 5, 15, and 60 min. The thermogram for the PHB sample obtained by dissolution in chloroform is shown as a control.

Figure 5a demonstrates a number of differences in the samples obtained by dissolution in acetic acid. The melting point of the PHB obtained by dissolution in chloroform was higher than those obtained by dissolution in acetic acid (175 °C vs. 157 °C for the PHB aa, respectively). This decrease in the melting point may be due to the formation of a less perfect crystalline component in the polymer. It should be noted that there is a small peak present in the thermograms of the PHB from the acetic acid around 125–140 °C. This peak can be attributed to the low molecular weight fraction resulting from the hydrolysis of the polymer. This effect has also been reported in the literature for this type of polymer [54]. The shift and bifurcation of the melting peak in the PHB sample that was in acetic acid for 60 min can be attributed to the large amount of low molecular weight fractions. The polymer’s structure is characterized by smaller, less perfect crystals that melt at lower temperatures when there is a higher proportion of low molecular weight fractions present [54].

The degree of crystallinity of the samples was calculated, revealing that those made with acetic acid had a lower degree of crystallinity, around 50% (PHB 0 min—50.8%, 5 min—50.8%, 15 min—50.6%, and 60 min—50.1%), compared to the PHB made with chloroform, which had a crystallinity of 61%. This is also in agreement with the literature data [54,55].

### 2.4. FTIR Spectroscopy

The next task was to study the structural features of the PHB samples obtained from the different solvents: acetic acid and chloroform (Figure 5b).

Both polymer samples were characterized by FTIR spectroscopy. The FTIR spectrum was recorded in the range of 4000–600 cm^−1^. Figure 5b shows that the spectra of the two samples were almost identical, with transmission bands at 1721 cm^−1^ observed, corresponding to the carbonyl (C=O) stretching of the ester. The band at 1453 cm^−1^ is attributed to the deformation of methyl (CH_3_) and methylene (CH_2_) groups. The bands at 1181, 1279, and 1228 cm^−1^ (not annotated) are sensitive to the crystallinity of the polymer. The bands at 1181 and 1128 cm^−1^ are characteristic of the asymmetric and symmetric stretching vibrations of the C-O-C group, respectively. The peaks at 979 cm^−1^ and 1721 cm^−1^ are characteristic of the vibrations of the corresponding groups in the crystalline phase of PHB [56,57,58,59].

A small peak at 1574 cm^−1^ is observable in the spectrum of the PHB aa. This peak can be attributed to the presence of acetic acid residues in the gel. Otherwise, the differences are minimal and only appear in the intensity ratio of the bands [56]. The FTIR results support the data that the use of acetic acid as a solvent does not affect the chemical structure of the polymer.

### 2.5. Degradation of PHB Gels

**Figure 6:**
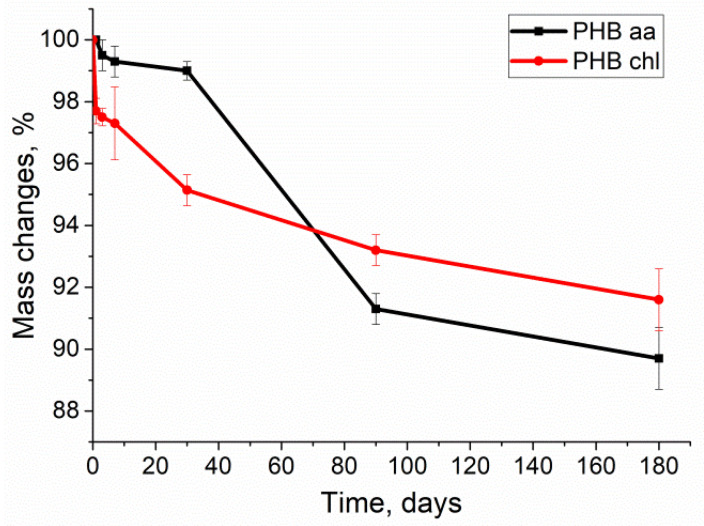
The change in the weight of the PHB during the degradation in the phosphate buffer in the presence of lipase.

One of the most important properties of poly(3-hydroxybutyrate) is its biodegradability, which largely determines its applicability in biomedicine. The results of the degradation of PHB samples in a phosphate buffer in the presence of porcine pancreatic lipase for 180 days are presented in Figure 6.

Figure 6 demonstrates that the degradation of PHB in a phosphate buffer in the presence of lipase is a slow process. By the 180th day of incubation, both samples had lost less than 10% of their initial mass. In comparison, the average degradation time of polylactide (PLA), the most commonly used biopolymer, is about 6 months [60,61]. A more pronounced mass loss can be observed for PHB chl on the first day of incubation when comparing the two curves. This phenomenon is also typical for PHB samples prepared in this manner and has been previously described [21,61,62]. A possible explanation is the relatively rapid evaporation of chloroform during the gel preparation. After the evaporation of the solvent, the supramolecular structures of the polymer are in a metastable state. In addition, there are regions of an amorphous component in the polymer matrix volume. When water enters the gel volume, it washes out the amorphous component and unbound polymer residues [50]. This is confirmed by the presence of nanometer-sized particles in the solution, as described in [61].When the material is obtained with acetic acid, no such effect is observed. This is likely due to the hydrolysis of all unbound polymer residues by the acid.

After 180 days of the experiment, the mass of the PHB aa decreased slightly more than that of the PHB chl (by 11% and 9%, respectively), as it is known that the degradation rate is influenced by the degree of the crystallinity of the polymer.

### 2.6. Scanning Electron Microscopy (SEM) of Dried Gels

**Figure 7:**
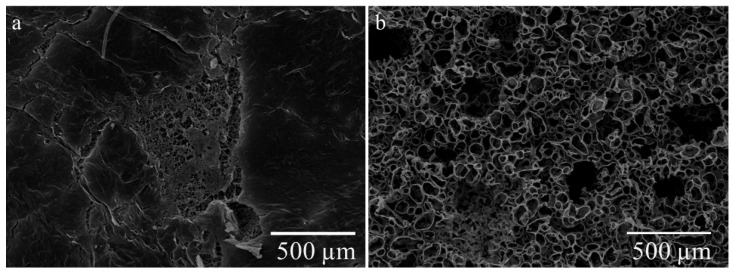
Surface image of PHB gels obtained from acetic acid (**a**) and chloroform (**b**).

SEM images of the PHB gel fabricated in the acetic acid and chloroform solutions are shown in Figure 7.

Figure 7 illustrates the significant differences in the microstructure of dried PHB gels prepared using different methods and solvents. The gel obtained by the leaching method (Figure 7b) contains numerous round pores and voids. Despite the presence of cracks, the surface morphology of the PHB aa from the freezing method appears more homogeneous than that of the PHB chl. Additionally, the sample surface of the PHB chl displays numerous pores with diameters ranging from 50 to 150 microns, which are likely related to the size of the pore-forming agent particles.

**Figure 8:**
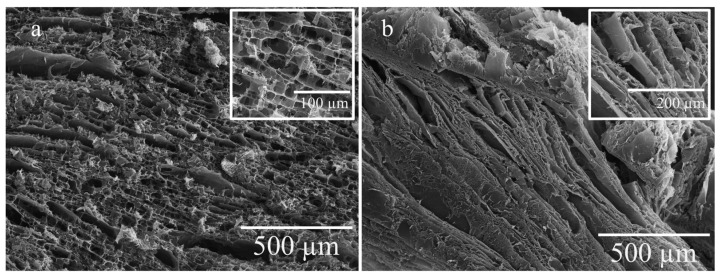
Internal structure of PHB aa gel: horizontal slice (**a**), vertical slice (**b**).

Thus, the peculiarity of the PHB aa is not the absence of pores, but the fact that most of the pores were hidden in the gel volume, since the cracks and fractures of the sample surface also show a well-developed porous structure (Figure 8a). In this case, we assume that the pore diameter varies considerably.

To further investigate the internal porous structure of the PHB aa, longitudinal and transverse slices of the gel were taken. SEM images of the PHB aa gels obtained from a horizontal slice (Figure 8a) and vertical slice (Figure 8b) are presented below.

Figure 7b and Figure 8a confirm the assumption that the pore size of PHB chl is significantly larger than that of PHB aa. In addition, a vertical pore structure was clearly observed for the PHB aa, which was delimited by long lamellae (Figure 8b). This is attributed to the freeze-drying process. Based on the literature data, the pore size and morphology influences the mechanical properties of the samples [63].Thus, the use of acetic acid as a solvent in the production of PHB-based gels also leads to the appearance of three-dimensional porous structures, as well as in the standard methods of the production of PHB gels.

### 2.7. Structure Parameters of PHB-Based Gels

To further quantitatively analyze the structure of PHB-based materials, parameters such as porosity and density were calculated. The results are presented in Table 1. In order to calculate the porosity, distilled water was employed as a pore-filling agent for the gel.

According to Table 1, the porosity of the PHB gels was sufficiently high in both cases, but the gels obtained using acetic acid were 5% more porous than those obtained by the conventional method. Also, the PHB aa gels had less density. According to the literature, materials with these density and porosity values can be used in tissue engineering structures. It is important to note that high porosity of gels promotes cell volume penetration and tissue growth [64,65,66].

The study of the swelling ratio for gels revealed that the PHB aa sample absorbed moisture over 50% more intensely than the PHB chl, which may also impact the materials’ biocompatibility.

### 2.8. Rheological Behavior and Stiffness of Gels

**Figure 9:**
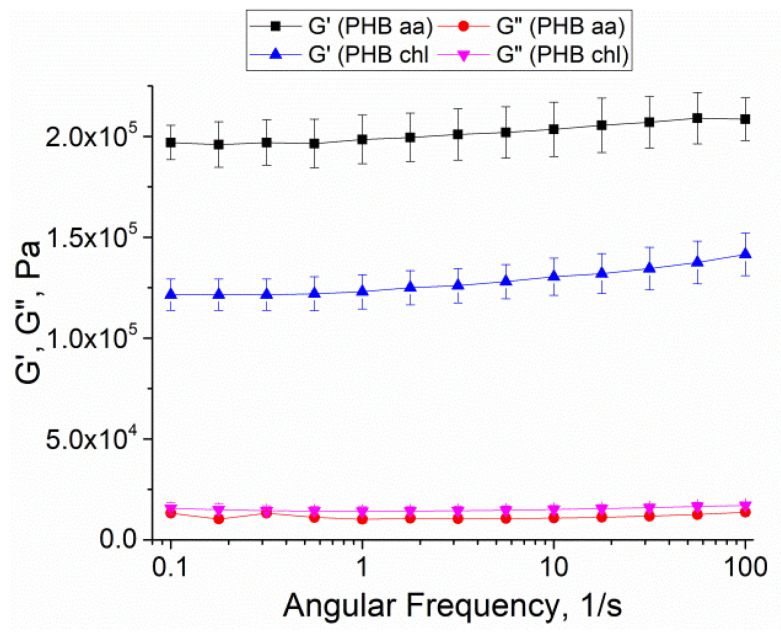
Storage modulus (G′) and loss modulus (G″) values for PHB gels.

The rheometry method was used to evaluate the mechanical properties of the fabricated gels. Measurements were taken in the phosphate buffer. Figure 9 shows the averaged data of the storage and loss moduli of the investigated samples.

The study on the mechanical properties using the rheometry revealed that the gels derived from acetic acid had a higher storage modulus (G′) compared to the PHB chl gels. The complex shear modulus (G*) values for the acetic acid-derived gels were statistically significantly different from those of the conventional method, as shown in Table 2. These differences suggest that PHB aa has a greater resistance to shear deformation. A potential explanation for these findings is related to the pore structure, which contains more junctions and bonds in the polymer thickness, potentially reinforcing the gel’s structure from within.

Young’s modulus is a mechanical property of gels that characterize their compressive stiffness. Our results demonstrated that the PHB aa gels were 2.4 times stiffer in compression experiments compared to the PHB chl. This difference is probably related to the internal structure of the gels. As can be seen from the SEM images, the pore size of the PHB chl sample was significantly larger than that of the PHB aa sample (Figure 8a). And based on the literature data, it is the pore size that has a great influence on the mechanical properties of materials [63]. In addition, a vertical pore structure was observed for the PHB aa, which was delimited by long lamellae (Figure 8b). They probably had a major influence on the stiffness of the framework and the increase in the mechanical properties.

The PHB chl gels had different pore sizes, which can create localized weakening in the overall gel structure. This is consistent with the shear tests and further confirms that the PHB aa had stronger mechanical properties, which is important for determining their practical applications.

### 2.9. Biocompatibility Tests of Gels

**Figure 10:**
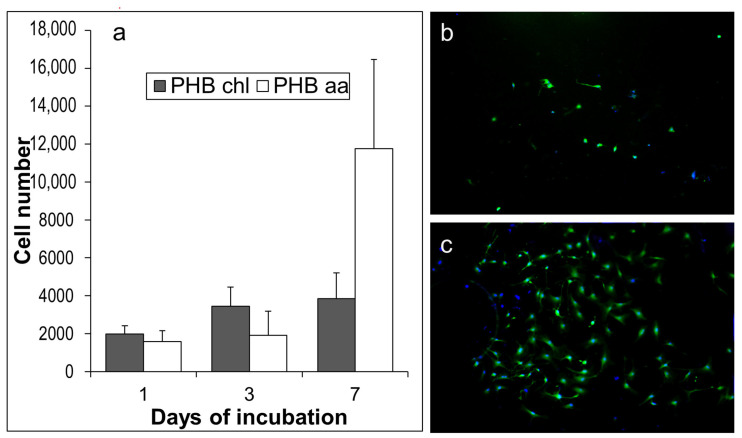
MSC growth on PHB chl and PHB aa at 1, 3, 7 days, Alamar Blue test (**a**); fluorescence micrograph of attached live MSCs on PHB chl (magnification ×100) after one day of incubation (**b**); fluorescence micrograph of attached live MSCs on PHB aa (magnification ×100) after one day of incubation (**c**).

The Alamar Blue test was used to evaluate the ability of cells to proliferate on the PHB samples. Figure 10a shows the degree of cell proliferation on substrates made of different materials.

The number of adherent live cells was also evaluated using fluorescence microscopy data, and the results are shown in Figure 10b,c.

No significant cell accumulation was observed on the PHB chl. Based on the Alamar Blue test data, which indicate the adhesion of approximately 2000 cells on the first day, it can be inferred that the cells do not adhere firmly enough to the surface and are easily washed off during sample preparation. The PHB sample derived from acetic acid showed a large number of widely spread cells, which is consistent with the results of the Alamar Blue test. The obtained results may be attributed to both the surface morphology of the studied samples and their material composition. For instance, even though PHB chl gel may contain only a small amount of chloroform, it can still have a toxic effect on cells. Additionally, there is evidence that the presence of low molecular weight PHB (for the PHB aa) can improve cell viability. This is due to the hydroxyl groups that are produced as a result of the breakage of ester bonds during acid hydrolysis [67,68].

## 3. Conclusions

In this study, a new method was proposed to produce PHB gels using acetic acid as an alternative and safer solvent. It was shown that glacial acetic acid hydrolyzes PHB upon heating, with the molecular weight loss ranging from 15% to 56% depending on the polymer dissolution time. We proposed a method of PHB dissolution in acetic acid with a minimal loss of polymer molecular weight, about 15%. On its foundation, 3D material (gel) was obtained by a freeze-drying method. The properties of the obtained material were compared with the properties of standard PHB material obtained from chloroform by a leaching method. It was found by DSC and FTIR that the structure of the materials was identical except for the presence of a small amount of low molecular weight components formed during hydrolysis in the PHB aa sample. However, for the PHB aa sample, a decrease in the PHB melting point and a lower degree of crystallinity was found compared to the PHB chl from 61% to 50.8%. The use of an alternative solvent for PHB insignificantly affected the enzymatic biodegradation process of the samples. After 180 days of the experiment, the mass of the PHB aa decreased slightly more than that of the PHB chl by 11% and 9%, respectively. The SEM study of the PHB-based gels in the different solvents showed differences in the microstructure and porosity of the samples, which influenced their viscoelastic properties. The porosity of the PHB aa was 97.7%, which was 5.2% higher than that for the PHB chl. In the rheological tests, it was shown that both the storage and loss moduli were higher for the PHB aa gel, which is also supported by the higher complex shear modulus for the PHB aa compared to that of the PHB chl, which were 198 kPa and 126 kPa, respectively. This indicates that the use of acetic acid makes the gels more resistant to shear stress as well as compressive stress. This fact was evidenced by the increase in compressive stiffness by 60% for the PHB aa. In vitro biocompatibility experiments showed that both samples were cytocompatible. The presence of low molecular weight PHB in the PHB aa sample had an effect on the MSC viability, expressed as a threefold increase in the number of attached live cells after 7 days of incubation compared to the PHB chl. In general, it can be concluded that the dissolution of PHB in acetic acid is quite promising for the development of biomedical materials. Acetic acid does not affect the main properties of PHB, but in some cases it will have a favorable effect on the characteristics of the 3D materials produced from it, especially structural and mechanical properties. However, in order to better realize the biomedical potential of the proposed PHB products using an alternative solvent, further testing is required to obtain 3D structures with a more controlled internal structure, as well as to modify the surface properties of the product to give it hydrophilic properties. This could be achieved by creating multi-component gels based on PHB aa, e.g., by mixing with inorganic substances.

## 4. Materials and Methods

### 4.1. Materials

Poly(3-hydroxybutyrate) (Biomer, Schwalbach, Germany), glacial acetic acid (Ekos-1, Moscow, Russia), chloroform (Ekos-1, Moscow, Russia), fetal calf serum (FCS) (Marlborough, MA, USA), DMEM, penicillin, streptomycin (PanEco, Moscow, Russia), Calcein AM (CCK-F) (DIA-M, Moscow, Russia), Hoechst 33258 (Servicebio, Wuhan, Hubei, China), and ammonium bicarbonate powder ((NH_4_)HCO_3_, Chimmed, Moscow, Russia) were used.

### 4.2. Preparation of PHB Dried Gels

#### 4.2.1. Preparation of Gels from PHB aa

The PHB pellets were pounded and placed in a glass flask. Then the glacial acetic acid was added to the flask. The concentration of the solution was 25 mg per mL of acid. The flask was placed on a magnetic stirrer and the reaction mixture was heated to 120 °C. As the mixture was heated and stirred, the PHB dissolved completely. The solution was then poured into a glass Petri dish, cooled in air, and then frozen and lyophilized. Before further experiments, all samples were neutralized with a 0.1 M sodium hydroxide solution, washed at least three times in distilled water to neutral pH, and then washed in ethanol.

#### 4.2.2. Preparation of Gels from PHB chl

The PHB gels in the chloroform were prepared by leaching, using a modified procedure. This modification was based on the temperature decomposition of the solid salt, whereas the standard method involves the solvent leaching of the salt. The polymer solution in the chloroform (25 mg mL) was mixed with ammonium bicarbonate powder (Chimmed, Moscow, Russia) in the ratio of 10:1 by weight and thoroughly mixed with a spatula. The mixture was placed in a glass Petri dish and left at room temperature until the organic solvent was completely evaporated (2–3 h). After that, it was placed in hot distilled water (70 °C) until gassing stopped, washed with distilled water 5 times, and dried at 37 °C for 24 h [34].

### 4.3. Molecular Weight Measurement

The molecular weight of the PHB samples obtained from both the chloroform and acetic acid (at different incubation times) was determined using the viscometric analysis method. Solutions of the polymers in chloroform and glacial acetic acid were prepared. For the acetic acid: at intervals of 0, 5, 15, 30, and 60 min, 2 mL of the PHB solution was taken and dried on glass until the solvent evaporated. The resulting sample was then dissolved in chloroform, and the molecular weight was determined using an Ubbelohde viscometer. The molecular weight was calculated using the Mark–Houwink–Kuhn Equation (1):

[η] = 7.7 × 10^−5^ × M^0.82^, (1)

where [η] is the characteristic viscosity [49].

### 4.4. Atomic Force Microscopy (AFM)

The structure of the PHB aa films during incubation in the acetic acid was studied using atomic force microscopy using an IntegraPrima instrument (NT-MDT SI, Moscow, Russia). Thin film samples were prepared by immersing freshly crushed mica in the polymer solution and air drying them. The samples were probed using NSG01 cantilevers (TipsNano, Moscow, Russia) with a stiffness of 1.45–15.1 N/m and a resonance frequency of 87–230 kHz in air in semi-contact mode. The images were acquired and analyzed using the Nova program (NT-MDT SI, Moscow, Russia).

### 4.5. Differential Scanning Calorimetry (DSC)

DSC thermograms were obtained using a differential scanning calorimeter DSC 204 F1 Phoenix (NETZSCH-Gerätebau GmbH, Selb, Germany). The study was performed in a nitrogen atmosphere with a flow rate of 40 mL/min and a sweep rate of 10°/min. The temperature interval was in the range of 25 to 200 °C. The degree of crystallinity was calculated from the second heating.

### 4.6. FTIR Spectroscopy

FTIR analysis of the samples was performed using a Spectrum Two FT-IR Spectrometer (PerkinElmer, Waltham, MA, USA) in the Attenuated Total Reflectance (ATR) mode. The following spectrometer characteristics were used: a LiTaO_3_ IR detector and a standard optical system with KBr windows for data acquisition in the spectral range of 4000–350 cm^−1^ with a resolution of 0.5 cm^−1^.

### 4.7. Enzymatic Degradation

The degradation of the materials was performed according to the methodology described previously [61,62]. Briefly, the dried gels were placed in a solution of lipase in a phosphate buffer at a concentration of 0.25 mg/mL. The solution was replaced with a fresh solution every three days. Sodium azide at a concentration of 2 g/L was added to the solution to prevent contamination. At the end of the incubation period, the samples were washed with distilled water, dried to a constant weight, and weighed on an Acculab-64 analytical balance (Sartorius (Acculab), Göttingen, Germany). Weight measurements were taken at 1, 3, 7, 30, 90, and 180 days. Three samples of each type were used for each control point.

### 4.8. Scanning Electron Microscopy (SEM)

The surface morphology of the gels was visualized by SEM using a scanning electron microscope JSM-6380LA (JEOL, Tokyo, Japan) and Tescan Vega 3 scanning electron microscope (SEM) (Tescan, Brno, Czech Republic) operated at low vacuum, at 10 kV (accelerating voltage), using a backscattered electron (BSE) detector. Gel samples were glued onto slides and gold plated using a vacuum atomizer. The prepared samples were transferred to the microscope chamber and images were acquired at 80 s/frame rate.

### 4.9. Determination of Swelling Ratio, Porosity and Density of Gels

The porosity and density of the gels were calculated in accordance with the methodology previously described in the literature [69]. Pre-weighed gels with a known mass were introduced to distilled water with a known volume. Water was then pumped into the gel, displacing air bubbles, using a series of air injection–pumping cycles. The total volume of the water and gel was subsequently measured. The gel was then removed from the water, and the remaining volume was measured. The porosity (ε) and density(d) were calculated using the equation used in [69].

The swelling ratio of the polymer gels was calculated using the standard method described in [70].

### 4.10. Rheological Tests

To evaluate the viscoelastic characteristics, samples of the gels with a diameter of 25 mm and a height of 3 mm were prepared. The rheological properties were investigated with an MCR-302 rheometer (Anton Paar GmbH, Graz, Austria) at 20 °C using a plate-to-plate geometry with a diameter of 25 mm. The dried gels were first placed in a PBS solution for 20 min. A frequency test was performed using a frequency sweep in the range of 0.1–100 rad/s with a fixed amplitude, and the values of the storage and loss moduli were determined. The value of the deformation amplitude was taken as 0.5%. The results were processed using OriginPro 2016 software (OriginLab Corporation, Northampton, MA, USA) [71].

In addition, Young’s modulus (compressive stiffness) was calculated. For this purpose, the normal force was plotted as a function of the displacement of the rheometer measuring geometry. The results were converted into stress–strain values. Young’s modulus was calculated from the linear region of the stress–strain curve.

### 4.11. Biocompatibility In Vitro Assay

For the biocompatibility study, sterilized samples were cut into 5 × 5 mm squares. A primary culture of mesenchymal stem cells (MSCs) isolated from the adipose tissue of 3-day-old Wistar rats was used. Cells were cultured on a DMEM growth medium with 4.5 g/L glucose (PanEco, Moscow, Russia) with the addition of 10% FCS (HyClone, Marlborough, MA, USA) and 50 U/mL of penicillin and 50 µg/mL of streptomycin solution (PanEco, Moscow, Russia). Sterile samples were seeded with cells at a concentration of 3000 cells/well in a 96-well plate. After 24 h, the degree of the cell adhesion to the samples was assessed by fluorescence microscopy by staining using calcein (2.7 μM) and Hoechst 33342 (3.8 μg/mL) in PBS for 30 min in a CO_2_ incubator at 37 °C. The stained cells were washed three times with PBS and analyzed using an Axio Lab.A1 fluorescence microscope (Zeiss, Jena, Germany). The images obtained were processed using the ImageJ program (Release 2.16.0) [72].

### 4.12. Statistical Processing of the Results

The statistical processing of the data was performed in OriginPro 2016 software (OriginLab Corporation, Northampton, MA, USAusing a one-way analysis of variance (ANOVA) and Student’s *t*-test at a significance level of *p* < 0.05. The data presented in the tables and figures are shown as ± SD unless otherwise specified.

## Figures and Tables

**Table 1:** Porosity, density, and swelling ratio for PHB aa and PHB chl.

Sample	Porosity, %	Density (d, g/cm^3^)	Swelling Ratio, %
PHB aa	97.7 ± 0.9	0.040 ± 0.009	200 ± 45
PHB chl	92.5 ± 1.0	0.077 ± 0.010	125 ± 10

**Table 2:** Mechanical parameters of gels.

Sample	Complex Shear Modulus (G*), kPa	Young’s Modulus, kPa
PHB aa	198 ± 12 *	101.5 ± 0.6 **
PHB chl	126 ± 8	41.3 ± 0.3

*, **—values differ significantly from PHB chl—*p* < 0.05.

## Data Availability

Data are contained within the article.
